# Quantifying the Impact of the Grain-for-Green Program on Ecosystem Health in the Typical Agro-Pastoral Ecotone: A Case Study in the Xilin Gol League, Inner Mongolia

**DOI:** 10.3390/ijerph17165631

**Published:** 2020-08-05

**Authors:** Zhaoyi Wang, Qianru Yu, Luo Guo

**Affiliations:** 1 College of the Life and Environmental Science, Minzu University of China, Beijing 100081, China; 16054039@muc.edu.cn (Z.W.); 18301244@muc.edu.cn (Q.Y.); 2 Department of Chemical and Biomolecular Engineering, National University of Singapore, Singapore 117570, Singapore

**Keywords:** ecosystem health, the Grain-for-Green program, agro-pastoral ecotone, Xilin Gol League, pressure-state-response model

## Abstract

The Green-for-Grain program (GGP) is the largest environmental restoration program in China. It is effective in controlling land desertification but at the same time is highly affected by regional differences. Ecosystem health, as an important indicator of ecosystem sustainability, can effectively assess the ecological impact of the GGP and provide a basis for follow-up actions. As a typical agro-pastoral ecotone along the Great Wall, the Xilin Gol League has seen increasing land-use intensity, thus, it is crucial to understand the ecological conditions of the region in order to deploy a policy of the GGP in accordance with local conditions. In this study, using remote sensing images and social statistics data from 1990–2015, land-use transformation and the turning point of vegetation coverage was determined. Based on the pressure-state-response (PSR) model, an ecological health evaluation system was constructed to quantify the temporal and spatial variation of ecosystem health. Then, the spatial correlation between the changes in forest and grass coverage, as well as the changes in the ecosystem health index (EHI), was evaluated using GeoDa software. The results showed that (1) grassland was the primary land-use/land-cover (LULC) in the Xilin Gol League. Since 2000, land-use transfer types changed significantly and grassland degradation weakened; landscape connectivity increased, and vegetation coverage increased. (2) Over the past 25 years, the ecosystem in the study area was at a subhealthy level and showed a trend toward a healthy level. (3) The spatial correlation between △Area% (change in forest and grass coverage) and △EHI (change in ecosystem health index) was positive between 2000 and 2015 and the correlation gradually increased, indicating that the GGP did enhance the health of the ecosystem of Xilin Gol. This study provided a specific reference for the evaluation of ecosystem health in the agro-pastoral ecotone of China and a theoretical basis for the implementation of sustainable management policies in the study area.

## 1. Introduction

The Grain-for-Green program (GGP) is one of China’s largest and best invested environmental projects [1]. Since 1999, China has gradually implemented ecological restoration projects centered around the GGP [2]. The GGP reduces soil erosion and increases carbon sequestration through increased vegetation cover [2,3]. Moreover, the GGP guarantees ecological sustainability and promotes harmonious development between humans and nature [4]. Although the GGP is one of the most effective measures in preventing and treating serious environmental problems [5], its effectiveness is limited by a number of socioeconomic factors, such as food production, local economic structure and farmer employment [6]. Some experts believe that the GGP is effective in improving ecosystem health [2]. Conversely, other experts argue that fencing natural grasslands reduces livestock farming and thus the livelihoods of pastoralists, which could affect the ecological and social development of the region [7]. Therefore, how to effectively assess the ecological impact of the GGP and how to formulate policy for the subsequent program according to local conditions is a key issue that China needs to solve immediately.

Ecosystem health is a comprehensive, multiscale measurement for ecosystem vigor, organization, and resilience [8]. A healthy ecosystem should be stable and sustainable [9]. However, ecosystems in a sick state are often in the process of gradually declining and irreversibly collapsing [10] with a high probability of problems such as soil erosion, desertification, or salinization of the land. Therefore, ensuring ecosystem health is seen as the foundation and driving force for environmental sustainability. As an important method of assessing ecological condition, quantifying ecosystem health through objective indicators provides us with clear goals for managing the complex system of the ecosystem [11]. There are two main approaches to quantify ecosystem health: the indicative species method and the indicator system approach [12]. The indicative species method requires a large amount of measured data (such as biomass, productivity, structural indications, and functional indications for endangered species, long-lived species, environmentally sensitive species, etc. of the ecosystem) on a species, whereas the indicator system approach is not limited by the number, type, and source of data on ecosystems [13]. The indicator system approach is generally used for ecosystem health assessment.

The pressure-state-response (PSR) is one of the most widely used models to guide the construction of the indicator system for ecosystem health evaluation [14]. PSR is based on the logical relationship between causes and consequences of problems and measures [15]. This model is often used to analyze selected environmental processes, identify relevant indicators, and ultimately provide a quantitative or qualitative description of causal relationships to understand the relationship between human activities and environmental impacts [16]. To address the shortcomings of the PSR model, namely, the subjectivity in indicator selection and weighting determination [17], we revised the model. First, we broadened the selection of indicators. The ecosystem service value was added in the evaluation indicators. In addition, the entropy weighting method was chosen to calculate weights to reduce human interference. More importantly, we used the PSR model in a 1000 m × 1000 m grid unit. Compared to previous studies of ecological health at the county level, we enhanced the analytical precision of PSR and it provided additional data for subsequent quantification of the effects of the GGP on ecosystem health.

An ecotone is a transition zone between adjacent ecosystems [18] where the temporal and spatial changes are rapid and that is sensitive to external changes [19]. The health of ecotones is important for biodiversity conservation, ecosystem management, and restoration and functional zoning of nature reserves. The Xilin Gol League, located in the middle of Inner Mongolia in China, belongs to the agro-pastoral ecotone along the Great Wall, and is an essential ecological barrier, boding a significant strategic position in China’s social and economic development [20]. The complicated terrain of the Xilin Gol League leads to plant growth difficulty. Furthermore, the rapid development of the population and economy in recent decades intensified the human–land conflict. In these circumstances, the ecological environment in the Xilin Gol League has severely deteriorated and gradually become one of the primary sources of wind and sand in North China. To solve environmental problems, the GGP was launched in the Xilin Gol League in 2000, which set five counties as project pilots. From 2000 to 2003, the whole region implemented the GGP project. To date, few studies had used the GGP as a control to assess temporal and spatial changes in ecological health. The analysis of the impact of the GGP on regional ecosystem health, however, is a key factor in understanding the ecological conditions of the region and developing the right reforestation measures. 

The purpose of this study is to (1) calculate the rate and direction of the land-use/land-cover (LULC) change in the Xilin Gol League by using the related land-use model; (2) use the PSR model to build an indicator system to evaluate the health status of the agro-pastoral ecotone in the Xilin Gol League between 1990 and 2015; (3) analyze the spatial correlation between the GGP program and the spatial and temporal changes in the health of the ecosystem health, aided by spatial analysis methods. The results provide data support and scientific guidance for the utilization of the ecosystem in the agro-pastoral ecotone and provide information regarding the sustainable development of agriculture and the follow-up to the GGP.

## 2. Materials and Methods

### 2.1. Study Area

**Figure 1:**
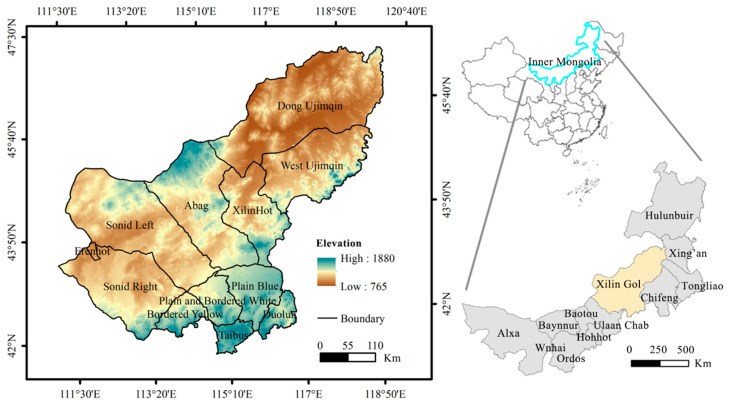
The location of the Xilin Gol League.

The Xilin Gol League (43°02′–44°52′ N, 115°13′–117°06′ E) is known as one of the four dominant natural grasslands in China; it borders the People’s Republic of Mongolia to the north (Figure 1). With a northern temperate continental climate, the Xilin Gol League features an average temperature of 0 °C to 3 °C, an average precipitation of 295 mm, an average relative humidity below 60%, and evaporation rates ranging from 1500 mm to 2700 mm. Annual sums of sunshine duration correspond to around 2800 h to 3200 h. The Xilin Gol League landform types include the shallow mountain/hill farming-pasturing interlaced zone, Hunshadake sandy land, and a degraded and desertified grassland zone. The Xilin Gol League covers an area of 2.026 × 10^9^ hm^2^, of which 1.796 × 10^9^ hm^2^ is grassland, 1.847 × 10^5^ hm^2^ is the arable land area, and 1.436 × 10^5^ hm^2^ is for the sowing of food crops. The permanent population of the Xilin Gol League is 1.055 million [21]. Agriculture is the main production source and grassland farming exists to supplement the agriculture.

### 2.2. Data Collection

The land-use data used for the study area were based on remote sensing images in the years 1990, 1995, 2000, 2005, 2010, and 2015 [22,23]. These images were produced by the Chinese Academy of Sciences Resource Environmental Data Center (RESDC) based on Landsat TM-/ETM+, Landsat 8 OLI and GF-2. According to Liu’s paper, they constructed a national land-use database for 1990–2015 via human–computer interaction using a high-resolution remote sensing–UAV–ground survey observation system, based on geographic knowledge [22]. The spatial resolution of land-use dataset is 1000 m × 1000 m. According to National Standard Land-Use Classification of China, land-use types in the study area are classified into 6 primary types and 13 subtypes. The primary classifications are farmland, forest, grassland, water, build and unused land. The comprehensive evaluation accuracy of the first level of land use is >93% and that of the second level is >90%, which can meet user mapping accuracy demands at a scale of 1:1 million [23]. Annual normalized difference vegetation index was based on the bimonthly dataset synthesized by Global Inventor Modeling and Mapping Studies (GIMMS) with a spatial resolution of 8 km (http://ecocast.arc.nasa.gov/data/pub/gimms/3g.v0/). The time series was from January 1990 to December 2015. The population data, per capita, and the annual average precipitation and temperature were derived from the Resource and Environment Data Cloud Platform, Institute of Geographic Sciences and Natural Resources Research, China Academy of Science (http://www.resdc.cn/). The spatial resolution was 1000 m × 1000 m. The data of soil erosion and wind and sand fixation from 1990 to 2015 came from the National Earth System Science Data Center. The spatial resolution was 1000 m × 1000 m. Socioeconomic data (i.e., total water resources and total agricultural output value) from 1990 to 2015 came from the Inner Mongolia Statistical Yearbook.

### 2.3. Methods

#### 2.3.1. Methods of Assessing LULC Changes

① Land-Use Dynamics Degree

The formula of the comprehensive land-use dynamic degree [24] is

(1) $$LC=\frac{\sum_{i=1}^{n}\Delta LU_{i\text{-}j}}{2\sum_{i=1}^{n}LU_{i}}\times \frac{1}{T}\times 100\%$$

where *LU_i_* is the area of *i*-th land-use cover at the beginning of monitoring (hm2); Δ*LU_i-j_* is the area of land-use cover *i* transforming to land-use cover *j* for the entire study region; *T* is the length of monitoring time (year).

② Landscape Pattern Index

In this paper, patch density (PD), the interspersion and juxtaposition index (IJI), patch richness (PR), the contagion index (CONTAG) and Shannon’s evenness index (SHEI) were selected. These indices can reflect the landscape structure [25]. The landscape pattern indices were analyzed by Fragstats 4.2 software [26].

③ LULC Change Direction Model

The LULC change direction model (LCDM) [27] is used to explore the direction and significance of LULC changes. The formula is

(2) $$LCDM=\frac{\sum_{i=1}^{n}\left[A_{\mathrm{ij}}\times \left(D_{J}-D_{I}\right)\right]}{A}\times 100\%$$

where *i* is the *i*-th land-use cover; *j* is *j*-th land-use cover transformed from the *i*-th in a specific period; *A*_*ij*_ is the area of *i*-th land-use cover transforming to *j*-th; *A* is the total transformed area of all land-use types in the entire study area during this period; *D_i_* and *D_j_* represent the ecological level of the LULC types before and after transformation (Table 1). A higher LCDM value indicates better ecosystem functions, whereas the lower the LCDM value, the more negatively the ecosystem is functioning.

#### 2.3.2. Assessing Ecosystem Health Based on the PSR Model

Based on the PSR model and taking the regional characteristics into account, the ecosystem health evaluation indicators were selected from the three layers of pressure, state and response [28]. The exact composition is shown in Table 2. The weights of the indicators were calculated by the entropy weighting method [29]. The raw judgment matrix [30] of the evaluation indicators was normalized using the extreme difference method. The formulae for calculating weight are as follows:

(3) $$w_{i}=\frac{1-H_{i}}{m-\sum_{i=1}^{m}H_{i}}(0\leq w_{i}\leq 1,\sum_{i=1}^{m}w_{i}=1)$$

(4) $$H_{i}=-k\sum_{j=1}^{n}f_{ij}\ln f_{ij},i=1,2,3,\ldots ,m$$

(5) $$f_{\mathrm{ij}}=\frac{r_{\mathrm{ij}}}{\sum_{j=1}^{n}r_{\mathrm{ij}}},k=\frac{1}{\mathrm{lnn}}$$

where *W_j_* characterizes the extent to which this indicator affects ecosystem health, *i* ∈ [1, *n*], *j* ∈ [1, *m*]; *i* is the *i*-th evaluation indicator, *j* is the *j*-th evaluation object; r is the statistical value of the *j*-th evaluation object on the *i*-th evaluation indicator.

Ultimately, the ecosystem health index (EHI) [31] was calculated. The range of EHI is from 0 to 1. For a more precise representation of the ecosystem health status, drawing on research results of experts [32] and combining the value of the ecological health index of the Xilin Gol League, the values of ecosystem health indices were classified into five levels: very healthy (0.8–1), healthy (0.6–0.8), sub healthy (0.4–0.6), unhealthy (0.2–0.4), and sick (0–0.2). The formula of the EHI model is as follows:

(6) $$EHI=\sum_{i=1}^{n}\omega_{i}\times b_{\mathrm{ij}}$$

where *EHI* reflects the ecosystem health index, *i* ∈ [1, n]; *i* is the *i*-th evaluation indicator; *w_i_* is the weight of the *i*-th indicator; *b_ij_* is the normalized value of the *i*-th indicator.

#### 2.3.3. Indicators of the PSR Framework

① Annual Normalized Difference Vegetation Index (NDVI)

To ensure consistency in the resolution of indicators, we fitted an Empirical Orthogonal Teleconnections (EOT) model [33] to resample GIMMS NDVI to a regular 1-km grid. According to Florian Detsch, the EOT algorithm performed reasonably well across space [34]. The maximum value composites (MVC) was used to synthesize monthly NDVI data. Based on monthly data, the average value of NDVI in the growing season from April to October was calculated as the annual NDVI using the mean value method [35]. The formulae are as follows:

(7) $$MNDVI_{i}=MAX(NDVI_{i1},NDVI_{i2})$$

(8) $$\bar{NDVI_{a}}=\frac{1}{7}\sum_{4}^{10}MNDVI_{i}$$

where *i* is the *i*-th month, *i* ∈ [4,10]; *MNDVI_i_* represents the maximum *NDVI* value of the *i*-th month; *NDVI*_*i*1_ and *NDVI*_*i*2_ represent the *NDVI* value for the first and second half of the *i*-th month; $\bar{NDVI_{a}}$ represents annual *NDVI* for year a, a ∈ {1990, 1995, 2000, 2005, 2010, 2015}.

② Ecological Resilience

Ecosystem resilience [36] was calculated from changes in vegetation type to determine the range of resilience forces or the size of the variable margin of an ecosystem. The formula is as follows:

(9) $$ER=\sum_{i=1}^{m}S_{i}\cdot P_{i}$$

where *ER* reflects the value of ecosystem resilience; *i* is the *i*-th land-use cover; *S*_i_ is the area ratio of each landscape type; *P*_i_ is the resilience value of the *i*-th landscape type (Table 3).

③ Ecosystem Services Value

Ecosystem services value is the benefits that humans receive directly or indirectly from an ecosystem. Based on the characteristics of the study area, drawing on the research experience of Li et al. [37], who improved Xie’s service value calculation formula applicable to the national level [38] and derived formulae suitable for calculating the ecosystem services value on regional scale, we decided to use the following formulae:

(10) $$ESV=\sum \left[A_{k}\times VC_{k}\times \frac{NPP_{s}}{NPP_{cn}}\times \left(\frac{2\times \frac{GDP_{\mathrm{ms}}}{GDP_{m}}}{1+e^{2.5-\frac{1}{En}}}\right)\right]$$

(11) $$v=\frac{1.05P\mathrm{re}}{\sqrt{1+(1+\frac{1.05P\mathrm{re}}{L}{)}^{2}}}$$

where *ESV* is ecosystem services value; *A_k_* is the area of land-use type k; *VC_k_* is the value of ecosystem services per unit area of land-use type k in China (yuan/ha) [37]; *NPP_s_* and *NPP_cn_* represent the net primary productivity of natural vegetation in the study area and in Cshina(t/ha/a); *GDP_m_* is China’s *GDP* per capita in 2002 (yuan/person); *E_n_* is the Engel coefficient for the study area in 2002; *v* is actual annual evapotranspiration (mm); *P*re is annual precipitation (mm); *L* is yearly mean evapotranspiration (mm).

#### 2.3.4. Assessing the Impact of the GGP on the EHI

In GeoDA software [39], Moran’s I [40] index is utilized to represent spatial correlation. It reveals the correlation between the values of a certain property of adjacent patches, ranging from −1 to 1. The bivariate local indicator of spatial association (LISA) displays the clustering of the two properties in every patch. Applied to the spatial analysis of ecosystem health, the spatial correlation pattern between △Area% (change in forest and grass coverage) and △EHI (change in ecosystem health index) can be shown by visualizing the distribution map and reveals the pattern of spatial differentiation of ecological health in the study area.

## 3. Results

### 3.1. Changes in LULC during 1990–2015

#### 3.1.1. Transformation Patterns of LULC

**Figure 2:**
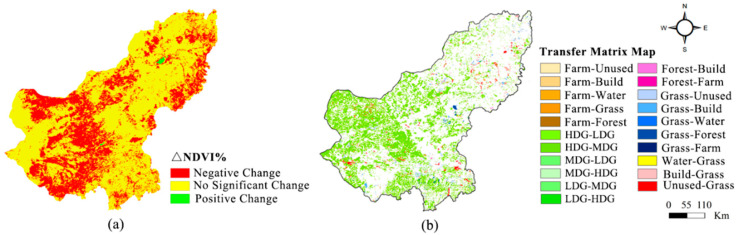
(**a**) Changes in the normalized difference vegetation index (NDVI) data from 1990 to 2015; (**b**) land-use transfer types from 1990 to 2015. (HDG: high-density grassland; MDG: mid-density grassland; LDG: low-density grassland).

NDVI was used to assess grassland productivity and quantify LULC changes. The NDVI in the Xilin Gol League featured a stripped distribution, which gradually declined from the east to the west. The annual NDVI for the entirety of the Xilin Gol League in 1990, 1995, 2000, 2005, 2010 and 2015 were 0.509, 0.499, 0.450, and 0.458, 0.473 and 0.456, respectively. Over the past 25 years, the trend of NDVI in the Xilin Gol League had been “gradually dropping–gradually rising–slowly dropping”. The NDVI dropped from 1990 to 2000 and, in 2000–2015, the vegetation gradually improved compared to the 1990s. Figure 2 presents the NDVI spatial changes in the Xilin Gol League from 1990 to 2015. Over the past 25 years, negative NDVI was concentrated in the southwest of the Xilin Gol League, where the most serious cases were the Sonid Left and Right Banner. Positive changes mainly appeared in Dong Ujimqin.

To evaluate the direction and rate of LULC change in the Xilin Gol League, we calculated the land-use dynamic degree and made a transfer matrix diagram (Figure 2). The trend of change in the land-use dynamic degrees between 1990 and 2015 was “rapid increase–rapid decrease–slow decline”. The land-use transition map revealed that the conversion between grasslands with different coverage was the most dominant land-use transfer type from 1990 to 2015. There was a significant increase in dynamic degrees after the implementation of the GGP. In terms of the spatial scope, grasslands transition was concentrated in the western part of the study area. The overall vegetation status in the study area started to take a turn for the better in 2000.

#### 3.1.2. The Directional Changes in LULC

The conversion between farm, forest, and grasslands is bound to affect ecosystem functions. The LULC change direction model was developed to assess directional changes in different LULC types. We calculated the LCDM values based on formula (2). The results are as follows: LCDM_1990__–1995_ = −0.0113%, LCDM_1995__–2000_ = 0.0255%, LCDM_2000__–2005_ = −0.1036%, LCDM_2005__–2010_ = 0.0084%, and LCDM_2010__–2015_ = 0.0590%. Between 1990 and 2015, the LCDM value showed an “up–down–up” trend. Negative values were recorded in 1990–1995 and 2000–2005. 

### 3.2. Changes in Landscape Patterns

The changes in the landscape structure of the Xilin Gol League from 1990 to 2015 are shown in Table 4. It could be seen that when the values of patch density (PD) and patch richness (PR) increased, Shannon’s evenness index (SHEI) decreased then increased. The values of the interspersion and juxtaposition index (IJI) decreased and then increased, while the contagion index (CONTAG) increased.

**Figure 3:**
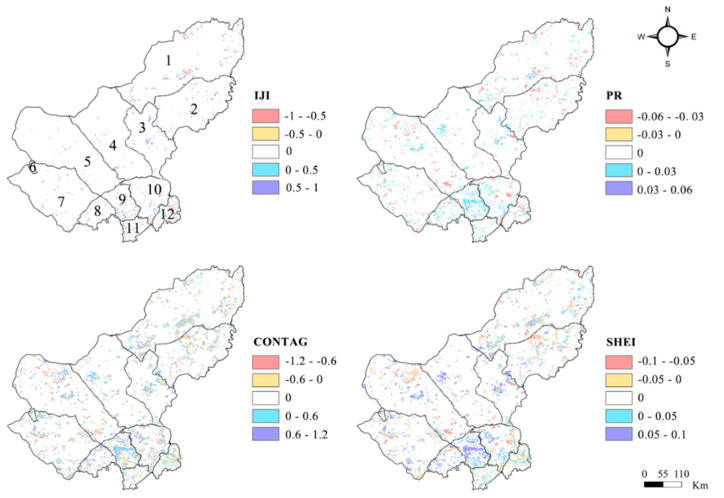
Spatial change in the landscape index in the Xilin Gol League between 1990 and 2015. (1: Dong Ujimqin; 2: West Ujimqin; 3: Xinlin Hot; 4: Abag; 5: Sonid Left; 6: Erenhot; 7: Sonid Right; 8: Bordered Yellow; 9: Plain and Bordered White; 10: Plain Blue; 11: Duolun; 12: Taibus Banner).

From 1990 to 2015, the spatial changes in the landscape pattern of Xilin Gol are shown in Figure 3. IJI, PR, and SHEI all increased in Plain and Bordered White and Taibus Banner, which is located in the south of the study area. In Sonid Right Banner and the Dong Ujimqin, there was no obvious change in IJI, while PR and SHEI decreased significantly. CONTAG increased in West Ujimqin, Sonid Right Banner, Duolun and Taibus Banner, and declined in Plain and Bordered White. Generally, IJI, PR, and SHEI changes were not visible, with slight rises in some areas. CONTAG fluctuated in most parts of the research area and the rising trend was more pronounced and the distribution wider. These plaques were mainly distributed in the south of the study area.

### 3.3. Changes in the Health of the Ecosystem during 1990–2015

#### 3.3.1. Temporal Analysis of Ecosystem Health

During 1990–2015, the ecosystem health index (EHI) in the Xilin Gol League ranged from 0.48 to 0.50 (Table 5). From 1990 to 1995, the study area showed a small increase in EHI values. From 1995 to 2000, there was a significant decline in EHI. During the decade 2000 to 2010, EHI values showed an upward trend until 2010, when the study area showed a small decrease in EHI values. However, the overall EHI of the Xilin Gol League increased from 2000 to 2015. Overall, the EHI in the study area showed an upward trend over the 25 years. Observing the calculation results of each standard level, the change trend of the total score in the pressure layer was “rising–falling–rising”, and the score of the Xilin Gol League increased over the 25 years. The total score in the state layer continued to decline in the early period, and it only picked up as of 2005. The total score in the response layer declined from 1990 to 2000 and began to rise in 2000. This was consistent with the start of the GGP in the study area.

#### 3.3.2. Spatial Patterns of Ecosystem Health

**Figure 4:**
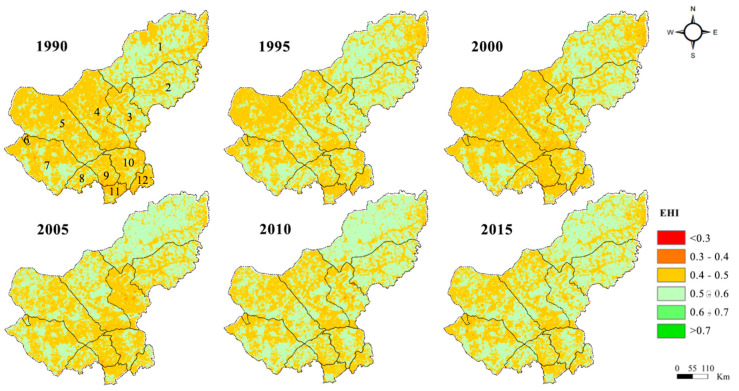
Ecosystem health index of the Xilin Gol League from 1990 to 2015. (1: Dong Ujimqin; 2: West Ujimqin; 3: Xinlin Hot; 4: Abag; 5: Sonid Left; 6: Erenhot; 7: Sonid Right; 8: Bordered Yellow; 9: Plain and Bordered White; 10: Plain Blue; 11: Duolun; 12: Taibus Banner)

The EHI spatial distribution was calculated based on the PSR model in the study area from 1990 to 2015 (Figure 4). The level of EHI was higher in the northeast of the Xilin Gol League than in the southwest. The change in the ecological health of the northeast was not significant. In contrast, a significant improvement occurred in the southeast when EHI increased in areas and decreased in areas with low EHI over time.

During 1990–2000, ecosystem health significantly deteriorated in the western part of the Xilin Gol League, especially in the Sonid Right Banner. From 2000 to 2015, the areas with a dropping EHI in the Dong Ujimqin Banner (located in the south of the Xilin Gol League), Sonid Left Banner, Sonid right Banner, Xilin Hot, and Bordered Yellow (located in the southwest of the Xilin Gol League) gradually expanded, meaning that the EHI of these league cities steadily dropped after 2000. However, the EHI of Dong Ujimqin Banner and Xilin Hot in the east of the study area improved when EHI values increased by more than the area where the EHI value decreased.

### 3.4. Impact of the GGP on the EHI

Univariate Moran’s I indexes for 1990, 1995, 2000, 2005, 2010, and 2015 were calculated as 0.279, 0.230, 0.226, 0.258, 0.274, and 0.169, respectively, which showed positive spatial correlation. To test whether Moran’s I was significant, Monte Carlo was used to simulate 999 tests in Geoda. All the results passed the significance test of 0.001, indicating that the spatial autocorrelation was significant at a 99.9% confidence level. From 1990 to 2000, Moran’s I index decreased, implying that the EHI spatial correlation gradually declined. From 2000 to 2010, Moran’s I index steadily increased and the EHI spatial distribution started to show a noticeable trend of clustering in the same direction. During 2010–2015, the EHI spatial autocorrelation in the study area suddenly declined.

**Figure 5:**
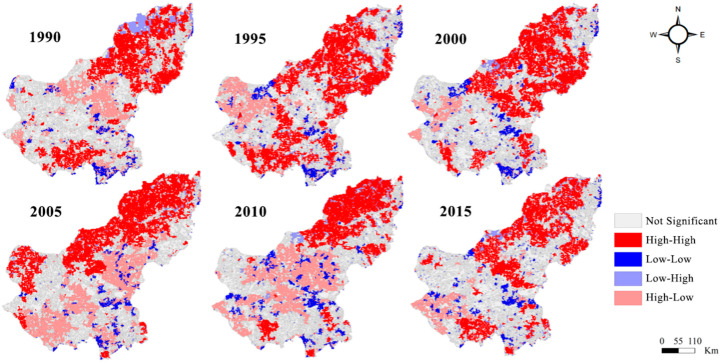
Significant areas of local Moran’s I for the ecosystem health index (EHI) of the Xilin Gol League from 1990 to 2015.

The local indicator of spatial association (LISA) (Figure 5) showed that high–high clustering (*p* ≤ 0.01) prevailed in the northern area of the study area. The most significant cluster appeared in Dong Ujimqin (*p* = 0.001). In the southwest of the Xilin Gol League, most of the areas showed insignificant correlation.

Bivariate local Moran’s I indexes for 2000–2005, 2005–2010, and 2010–2015 were 0.183, 0.270, and 0.313, respectively, which meant that △EHI (change in ecosystem health index) was positively spatially correlated with △Area% (change in forest and grass coverage), and the correlation strengthened within 15 years of the GGP implementation. In the correlation between △Area% and △EHI in 2005–2010, the Dong Ujimqin Banner, West Ujimqin Banner, and Abag showed a high–high correlation (*p* = 0.05), while Xilin Hot showed low–high correlation and the correlation in other counties were not significant. In 2010–2015, Dong and West Ujimqin Banner showed a high–high correlation (*p* ≤ 0.05), XilinHot showed low–high correlations and the significance increased (*p* = 0.01). Particularly, a significant low–low correlation appeared in Plain and Bordered White (*p* = 0.001).

## 4. Discussion

### 4.1. The GGP Changed the Transfer Direction of LULC

Grassland was the main LULC type in the Xilin Gol League, constituting 83.87% of the total LULC from 1990 to 2015 and existing mainly in the form of high and medium coverage. Furthermore, while forest and arable land gathered in the northeast and south of the study area, the utilized land was scattered among the grassland, and the construction land was interspersed among the arable land. Since the implementation of the GGP in 2000, apparent changes have occurred in the Xilin Gol League’s land-use, with arable land slowing down the pace of increase, and the area of grassland changing from a rapid decline to a slow deterioration and even a rise. As for land-use transition types, the transition between different grassland types was the primary change type in the 1990s. After 2000, the transition within different grassland types gradually decreased and the transition of arable land and unutilized land appeared. In terms of the spatial scope, the grassland change areas were concentrated in the west of the study area and the transition distribution of the unutilized area was extensive. Analyzing the changes in land use from the perspective of landscape pattern, the changes in SHEI and IJI values showed a turning point in 1995, indicating that the landscape diversity and connectivity in the study area have declined. However, this situation has gradually improved since 2000.

According to NDVI analysis of the Xilin Gol League from 1990 to 2015, the vegetation coverage of the study area changed in 2000, which could be attributed to reclamation. By comparing the distribution and area changes in NDVI in different thresholds over the past 25 years, we confirmed that vegetation coverage changed. The vegetation growth worsen from 1990 to 2000, while in 2000–2015, the vegetation gradually improved. Combining relevant policies, the change mainly benefited from the GGP in the study area since 2000. A significant influence of the implementation of the GGP increased vegetation coverage. Over recent decades, climate conditions in the study area have been stable. The LULC changes were consistent with the time nodes and spatial distribution of NDVI changes. Therefore, we concluded that NDVI changes were mainly caused by reclamation.

The changes in LULC and NDVI indicated that the policy of the GGP in the Xilin Gol League was promoted step-by-step and region-by-region between 2000 and 2015. From 2000 to 2005, the study area banned grassland restoration to effectively curb grassland reclamation. During this period, forest and grass coverage annually increased and areas with a low value reduced. Forest and grass coverage in most areas were at a medium level. From 2005 to 2010, the transition of arable land and unutilized land to grassland appeared, and grassland increased by 535.8 km^2^, suggesting a significant increase in forest and grass coverage. Areas with high NDVI reached their most senior over 25 years. From 2010 to 2015, the implementation of the GGP loosened. At the same time, the dynamic degrees of land utilization was only 0.6027%. In small areas of the southwest, high coverages of grassland were replaced by low grassland coverage and the annual average NDVI decreased. LCDM results from 1990 to 2015 showed that LULC changed and was affected by the return of arable lands to forests and grasslands. The gradual increase in LCDM values after 2005 suggested that LULC changes had beneficial effects on ecosystem functions in the Xilin Gol League, providing preliminary evidence of the positive effects of the GGP on ecosystem health.

### 4.2. The GGP Enhanced the EHI of the Xilin Gol League

The results of ecosystem health showed that the Xilin Gol League ecosystem remained in a sub-healthy state. In general, the health of the ecosystem of the study area improved (Table 5). The increase in the total value of the pressure layer proved that with the development of economy and society, population pressure and economic pressure were increasing. At the same time, with the global resource shortage and climate deterioration, the land and water resources in the study were gradually decreasing. Analyzing the results of the state and response layer, the improvement effect of the GGP launched in Xinlin Gol in 2000 on the function of regional ecological structure only began to appear in 2005, while the government and the people’s response was immediate. The *W*_j_ value calculated by the entropy method can characterize the impact of the indicators on ecosystem health. The higher the value of *W*_j_ was, the more significantly the indicator impacted ecosystem health. It can be seen from Table 2 that the *W*_j_ values of arable land area per capita and grassland area per capita were the largest in the pressure layer, indicating that these two indicators had a major impact on ecological health. Both are processed based on LULC data with a resolution of 1 km, indicating that unreasonable land-use was the greatest pressure exerted on the ecosystem and the main driving factor to reduce EHI.

Moran’s I index for all six periods showed positive values around 0.25, indicating that the spatial distribution of EHI values did not exhibit complete randomness, but rather there was some degree of clustering. Across the study area, large EHI areas were highly–highly clustered (Figure 5), suggesting that the spatial correlation in the Xilin Gol League was characterized with higher EHI areas adjacent to each other. This meant that a healthy ecosystem could drive the surrounding ecosystem in a positive direction. From 1990 to 2000, the number of not-significant-type areas increased and gathered in the southern part of the study area, indicating that the ecosystem in the study area in the 1990s tended to be fragmented, and spatial differences in EHI emerged. From 2000 to 2010, the percentage of areas featuring the high–high clustering rose to 9.58%, which suggested that more and more areas within the Xilin Gol League tended to be healthier in recent years. These areas had also driven the health of the surrounding areas.

In the 1990s, the health of the ecosystem gradually worsened because large areas of land were transitioning to arable lands in order to develop the local agriculture, which was adapting to population growth. The forest and grass coverage also dropped, leading to increased pressure in the research area. The worsening areas of ecological health were most obvious in Dong Ujimqin Banner and Duolun County, where arable lands were concentrated. From 2000 to 2010, the GGP was comprehensively implemented in the Xilin Gol League, which effectively alleviated grassland degradation. The decreasing per capita arable land area at the pressure layer and increasing NDVI at the state layer were mainly attributed to the rising EHI of the study area over 10 years. The state of health deteriorated from 2010 to 2015 because the Xilin Gol League failed to vigorously implement the GGP, which resulted in an increasing reclamation rate of lands and a decreasing NDVI. The degradation of grasslands with high and medium coverage in Sonid Right Banner increased plaque density, decreased landscape diversity, and destabilized the study area’s system structure.

With the launch of the GGP, EHI changes and areas of forests and grasslands were in direct proportion to each other. The correlation strengthened annually. After the implementation of the GGP, large areas of arable land and unutilized land were transferred to forest and grassland, leading to an increasing landscape abundance of the forest and grass ecosystem, and the interconnection of adjacent plaques. This meant that the GGP improved the health state of the ecosystem in the research area. The Xilin Gol League turned farmland to forest in 2000, and in 2003, turned farmland to grasslands. In the late period of the GGP project (2010–2015), emphasis on the GGP implementation weakened, resulting in a recovery speed slowdown of forestland and grassland. In five years, the EHI demonstrated negative changes in the whole area and was responsible for the spatial relations of the low–low clustering area between the forest and grass coverage changes and the EHI indices in the clustering diagram.

### 4.3. Recommendations for the Sustainable Development of Agro-Pastoral Ecotone

In areas with relatively poor ecological conditions, it is necessary to adopt effective measures to curb the trend of grass degradation and realize the balance among “humans, livestock, and grass” by protecting grassland environmental security. The environmental protection red line of grassland resources should be demarcated to protect the grassland areas and types within the environmental protection red line. Abuse of grassland resources should be banned to ensure the sustainable development of grasslands. As for other areas, protection of grassland with high and medium coverage should also be strengthened to prevent the regression of grassland to low coverage. Grassland should be restricted from being reclaimed into arable land. The GGP should be carried out on wasteland without too much utilization value. The GGP should be tailored to different geographical locations, climatic, and stand conditions. There should no longer be a limit on the ratio of restored ecological grasslands to economic grasslands. The focus should shift to increasing vegetation cover and mobilizing peasantry enthusiasm, making the GGP a conscious action of the general public to protect the ecological environment and improve production. Therefore, a subsidy policy may be established based on the prescribed task of the GGP. The local government should strengthen the advertising among the pastoralists and enhance herdsmen’s awareness of the social, economic, and cultural values of grassland resources, as well as the role of grassland in protecting the environment. The local government should also improve the operation means and economic efficacy of the animal husbandry to gradually upgrade the traditional internal value of grassland to landscape ecology. Meanwhile, the local government should enhance the monitoring of grassland resources and improve grassland investment mechanisms.

We propose the following management measures according to the EHI of the Xilin Gol League:

The basic grassland types in the agricultural area of Siqi County, located in the south of the Xilin Gol League, should be divided. Taipusi Banner, Zhengxiangbai Banner, Sonid Right Banner, and Zhenglan Banner should refer to the advanced experience of Duolun County with favorable ecosystem health in order to finish and subcontract the grassland division. This would guarantee personnel, equipment, and funds, as well as synchronize on-the-spot dotting and information collection, and standardize archive management.

The non-shepherding project should be launched in the north of the Xilin Gol League. The shepherding area should be strictly controlled in ecologically vulnerable areas such as the Ujimqin Sand to gradually realize the withdrawal of sheep raising in the whole area. The grassland ecological subsidy and reward policy should be launched to strictly control the grazing capacity and the scale of grazing in winter on natural grassland. The issuance of subsidies and rewards should be linked with the responsibilities of agricultural and pastoral households to ensure the realization of the ban on shepherding in the shepherding area and also to understand equilibrium in the equilibrium area.

Xilin Hot is the seat of the Xilin Gol League government and is situated precisely to the south of Beijing. Xilin Hot governs the environmental civilization construction of the Xilin Gol League. It is necessary to strengthen problem awareness and objective orientation, constructing a better environmental security barrier. Major environmental engineering projects, such as the Beijing-Tianjin Sandstorm Source Phase II Project, the Wulagai River Water Conservation Forest Phase II Project, and other major environmental engineering projects should be implemented to protect grasslands. Meanwhile, specific implementation plans or regulations should be promulgated to guide departments to lower levels of grassland management.

The environmental protection red line of the whole Xilin Gol League area should be completed. Representative areas should be chosen from the north, center, and south of the Xilin Gol League, including Dong Ujimqin Banner, Sonid Left Banner, and Plain Blue Banner. These three areas should be selected as pilot areas for red line demarcation and boundary settlement. The grassland dynamic detection and evaluation should be strengthened, and the strictest control system should be established to realize non-grazing in the non-grazing area and prevent overload in the equilibrium area.

## 5. Conclusions

In order to better achieve ecosystem restoration and healthy ecosystem development in agro-pastoral ecotone, in this study, using the Grain-for-Green program (GGP) as a control for the temporal and spatial variation of ecological health indices, we assessed the process of the reforestation project and its impact on the ecological health of the agro-pastoral ecotone to propose more locally appropriate policies for reforestation and restoration. When evaluating the ecological health of the study area, the PSR model was modified to address its shortcomings. We adopt the principle of large-scale and multifactor indicator selection to improve the accuracy of the results with innovatively introducing ecosystem service values in the state layer. The entropy weighting method was used to calculate the weights, eliminating human interference. During the period of 1990–2000, land-use change was characterized by the reclamation of cultivated land and degradation of grasslands. The NDVI gradually decreased and the landscape tended to be fragmented, while it was effectively controlled during the period of 2000–2015 (after the implementation of the GGP). The study area had been in a subhealthy state for the past 25 years, with high EHI values mainly found in areas with high forest and grassland cover, such as the northern part of the Xilin Gol League. In response to the GGP, the rational use of land led to a decrease in grassland degradation and an increase in vegetation cover in the Xilin Gol League. From 2000 to 2015, the rate of change in forest and grassland area was positively correlated with the change in EHI value, and the area of high–high clustering was dominant, proving that the correct implementation of the policy of returning farmland to forest can effectively improve the ecological health of the mixed farming and pastoral areas. The spatial distribution of EHI was largely influenced by land-use. Up to now, it can be seen that the GGP was carried out in a step-by-step and zoned manner in the Xilin Gol League. Initial results were achieved in reforestation and combating desertification. In recent years, however, it can be seen that the policy of the GGP has slackened. Thus, more attention should be paid to achieving a high degree of integration of ecological, economic and social benefits while at the same time maintaining and consolidating the existing ecological benefits, so as to achieve sustainable development of the regional economy. Reasonable land-use practices are crucial for the sustainable development of the agro-pastoral ecotone. Understanding the rational and efficient use of land resources and developing relevant environmental policies are key issues for the future.

## Figures and Tables

**Table 1:** Ecological level of different land-use [27].

Land Use	Farm	Forest	Shrubbery	HDG	MDG	LDG	Water	Unused
ecological level	0.110	0.245	0.147	0.125	0.063	0.018	0.282	0.010

HDG: high-density grassland; MDG: mid-density grassland; LDG: low-density grassland.

**Table 2:** Index system of the ecosystem health evaluation and their weights.

Target Level	Standard Level	Feature Level	Indicator Level	Weight
EHI (Eecosystem Health Index)	Pressure	Population	Population Density	0.0530
			Population Growth Rate	0.0519
		Economy	GDP per capita	0.0547
		Resources	Land Reclamation Rate	0.0545
			Arable Land Area per capita	0.0604
			Grassland Area per capita	0.0604
			Water Resources per capita	0.0519
		Climate	Annual Precipitation	0.0457
			Annual Average Temperature	0.0502
	State	Vigor	Forest and Grass Coverage	0.0487
			Annual Normalized Difference Vegetation Index	0.0470
		Organization	Shannon’s Diversity Index	0.0672
			Average Patch Area Index	0.0662
		Resicilence	Resicilence	0.0663
		Contribution	Ecosystem Service Value	0.0759
	Response	Nature–Ecology	Soil Erosion	0.0474
			Windbreak and Sand Fixation	0.0465
		Society–Economy	Proportion of Agricultural and Animal Husbandry Output Value	0.0521

**Table 3:** Ecosystem resilience coefficient of different land-use [36].

Ecosystem Type	Forest	HDG	Paddy Field	Dry Land	Water	MDG	LDG	Build	Unused
P_i_	8.5	7	6.5	6	4	3.5	2	0	−2

R: ecosystem resilience; HDG: high-density Grassland; MDG: mid-density grassland; LDG: low-density grassland.

**Table 4:** Changes in the landscape pattern index in the Xilin Gol League.

	PD	IJI	PR	CONTAG	SHEI
1990	0.17	3.93	1.46	11.75	0.31
1995	0.17	1.58	1.46	11.76	0.12
2000	0.17	4.03	1.47	11.78	0.31
2005	0.17	4.29	1.49	12.17	0.32
2010	0.17	4.29	1.48	12.18	0.31
2015	0.17	4.64	1.49	12.30	0.32

PD: patch density; IJI: interspersion and juxtaposition index; SHEI: Shannon’s evenness index; PR: patch richness; CONTAG: contagion index.

**Table 5:** Results of ecosystem health assessment in the Xilin Gol League from 1990 to 2015.

	1990	1995	2000	2005	2010	2015
Pressure	0.22	0.23	0.23	0.22	0.23	0.24
State	0.19	0.18	0.18	0.18	0.18	0.18
Response	0.08	0.07	0.07	0.09	0.09	0.08
EHI	0.49	0.49	0.48	0.50	0.50	0.50
